# Bariatric Surgery and Weight Gain: Bibliometric Analysis

**DOI:** 10.1007/s11695-024-07055-1

**Published:** 2024-01-16

**Authors:** Damla Seckin, Fatma Cebeci

**Affiliations:** 1 Nursing Faculty, Akdeniz Universitesi, Kampus, Antalya, 07058 Turkey; 2 Nursing Faculty, Surgical Nursing Department, Akdeniz Universitesi, Kampus, Antalya, 07058 Turkey

**Keywords:** Weight regain, Bariatric surgery, Obesity, Bibliometric analysis

## Abstract

**Purpose:**

Bariatric surgery is the most successful method for weight loss; however, weight regain may occur in the long term. It depends on eating habits and self-management. The study aimed to conduct a bibliometric analysis on bariatric surgery and weight gain and to determine the content and trends in the literature.

**Materials and Methods:**

The scan was performed using the keywords “bariatric surgery” and “weight gain” in the Web of Science database. The years of publications and citations, the distribution of publications according to journals, research areas, and countries, co-authorship, co-occurrence, and co-citation were analyzed. The VOSviewer program was used for the analysis. Grey literature, books, and book sections were not included.

**Results:**

A total of 988 articles were included. The results showed that the most published and cited journal was *Obesity Surgery*, and the most published country was the USA with 313 publications. The most commonly used keywords were “bariatric surgery,” “obesity,” and “weight regain.” Harvard University was the most publishing institution with 50 publications. The most published year was 2022 since 1993 (n:118).

**Conclusion:**

In the study, current publications and research trends related to bariatric surgery and weight gain were analyzed through bibliometric analysis. It was concluded that weight regain after bariatric surgery should be focused on since it adversely affects the life of individuals, reduces the probability of success of surgical treatment, and imposes additional burdens on the healthcare system.

**Graphical Abstract:**

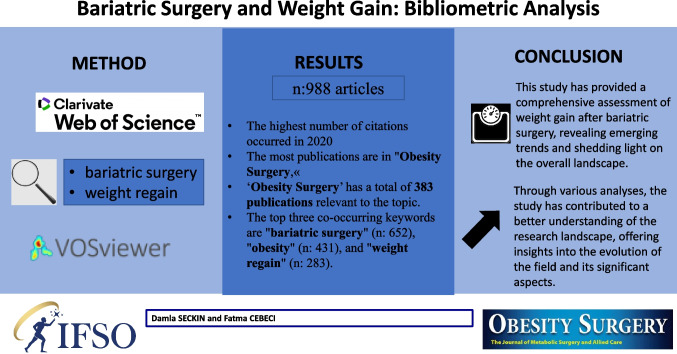

## Introduction

Today, bariatric surgery is considered the most effective treatment for obesity [1]. Maintaining weight loss after bariatric surgery is very important, and this process mostly depends on individual factors. Patients who have difficulty maintaining weight loss and realizing their self-management can gain weight in a long period [2–5]. Studies show that patients losing 20–30% of their total weight within a year or two after surgery will regain 7% of the lowest weight averages they have reached in 10 years [6–8]. In a systematic review based on a 7-year follow-up of patients with sleeve gastrectomy, it is stated that 37% of patients have gained significant weight [4]. Another study highlights that 36% regain weight within 5 years after surgery [9].

Weight regain after bariatric surgery is a serious problem closely associated with the negative impact on the patient’s physical health, psychological well-being, and the success of the surgery. Determining weight regain after bariatric surgery is a crucial factor for improving and maintaining the patient’s health, controlling physical health, enhancing quality of life, safeguarding psychological and mental health, and identifying surgical complications and risks [10].

In recent years, one of the main reasons for the increase in weight regain after bariatric surgery is that patients focus solely on weight loss related to the surgery. Patients often neglect the responsibilities related to lifestyle changes that are necessary for the surgery. As a result, at the end of this process, patients either experience insufficient weight loss or regain weight. Another reason is the preparation and support of the patient during the surgery process. Patients need to know what lifestyle changes they need to make after the surgery and how to implement them. Patients should be well-prepared in this regard. In cases where this preparation and environmental support are insufficient, patients may revert to their old habits and gain weight again. Another factor leading patients to weight gain is emotional hunger. Patients often experience emotional hunger in the postoperative period, leading to unconscious eating behaviors in various situations. Finally, in rare cases, patients may gain weight due to surgical problems [11–14].

Although the importance of lifestyle changes for patients undergoing bariatric surgery is emphasized, their long-term adaptation to these changes is often weak. The most common causes of weight gain include genetic factors, hormonal disorders, diet-related factors (such as uncontrolled eating and grazing), poor adherence to nutritional recommendations, reverting to preoperative eating habits, sedentary lifestyle, hypoglycemia, use of obesogenic drugs [15], and psychological factors (such as depression) [7, 16–23]. Weight gain related to surgical and anatomical factors (such as dilated gastric fundus, enlargement of the gastric pouch) is also observed [7, 15, 19, 24].

There are behavioral, nutritional, and pharmacological recommendations for preventing postoperative weight gain. Behaviorally, cognitive-behavioral therapy, various behavioral interventions, and lifestyle counseling are suggested [25]. Nutritional counseling with a dietitian is recommended for dietary aspects. Pharmacologically, FDA-approved drug treatments are recommended. Additionally, factors such as advanced age, male gender, preoperative high BMI, psychological problems, and the presence of comorbid conditions (type 2 DM, hypertension, and sleep apnea) are factors that increase the likelihood of postoperative weight gain [7]. Considering these factors, personalized patient follow-up services should be provided.

Bibliometric research is conducted to reveal the intellectual structure and evolutionary development over time and to define the productivity in the field [26]. In the literature, bibliometric studies on bariatric surgery are mostly related to current surgical procedures, publication trends, and comorbid diseases. Therefore, there are numerous publications in the literature addressing weight regain after bariatric surgery, reflecting a variety of perspectives and insights. However, no bibliometric analysis study has been encountered regarding the determination of trends and needs in this field. It is believed that our study will fill the gap in this field. The study aims to define topic clusters related to weight regain after bariatric surgery and to highlight the conceptual development in the field throughout the historical process. This research intends to provide a general assessment of the weight regain in bariatric surgery, identify emerging trends, determine the authors, journals, and institutions contributing most to the field, and analyze article contents and trends.

## Method

### Research Purpose

The study aimed to define the subject clusters with bibliometric analysis on weight gain after bariatric surgery, to reveal the conceptual development, and to determine the content and trends.

The following questions were considered in this research.

- What is the annual publication rate of weight gain articles in bariatric surgery?
- Which are the most prolific authors, countries, institutions, and journals on weight gain and bariatric surgery?
- What are the results of the citation analysis related to weight gain and bariatric surgery?
- What are the most common words and keywords about weight gain and bariatric surgery?

### Design

This study employed a descriptive bibliometric research design.

### Data Collection

Web of Science (WoS) was used as the data source. It is the most widely used database in bibliometric studies and includes journals indexed by Science Citation Index (SCI), Science Citation Index-Expanded (SCIE), Social Science Citation Index (SSCI), and Art, and Humanities Citation Index (A&HCI). It consists of publications, compilations, proceedings, and book sections of selected journals. Informed consent does not apply.

### Scanning Strategy

The data scan was carried out on November 13, 2023, on the Web of Science database using the keywords “bariatric surgery” and “weight gain” without year limitation. Derivatives of these words were also included. The words were given in the following table (Table 1).

**Table 1:** Scanning terms

	Keywords
Words related to weight gain	TS = (“body weight change”) OR TS = (“weight gain”) OR TS = (“weight regain”)
Words related to bariatric surgery	(((((((TS = (“bariatric surgery”)) OR TS = (“metabolic surgery”)) OR TS = (“gastric bypass”) OR TS = (“sleeve gastrectomy”) OR TS = (“gastric band”) OR TS = (“biliopanctreatic diversion”) OR TS = (“duedonal switch”) OR TS = (“intragastric balloon”)
Document type	Article
Language	English

### Inclusion and Exclusion Criteria

Articles published in English were included while early access articles, review articles, papers, editorial articles, grey literature, books, and book chapters were excluded. Duplications were determined.

### Data Collection

A bibliometric analysis of the studies evaluating weight gain in bariatric surgery was performed without time limitation. Articles in different disciplines and fields were included. The data were examined through author, citation, journal, country, institution, keyword, and abstract analysis. The researchers evaluated the results of independent scans and included studies meeting the inclusion and exclusion criteria.

### Methods of Analysis

For bibliometric analysis, the scientific mapping tool “VOSviewer” was used, providing better visualization in network and cluster analysis. In the analysis, journals, countries, institutions, cited publications, authors, co-occurrence network analysis of keywords, word analysis of abstracts, and co-citation analysis were performed.

## Results

**Fig. 1:**
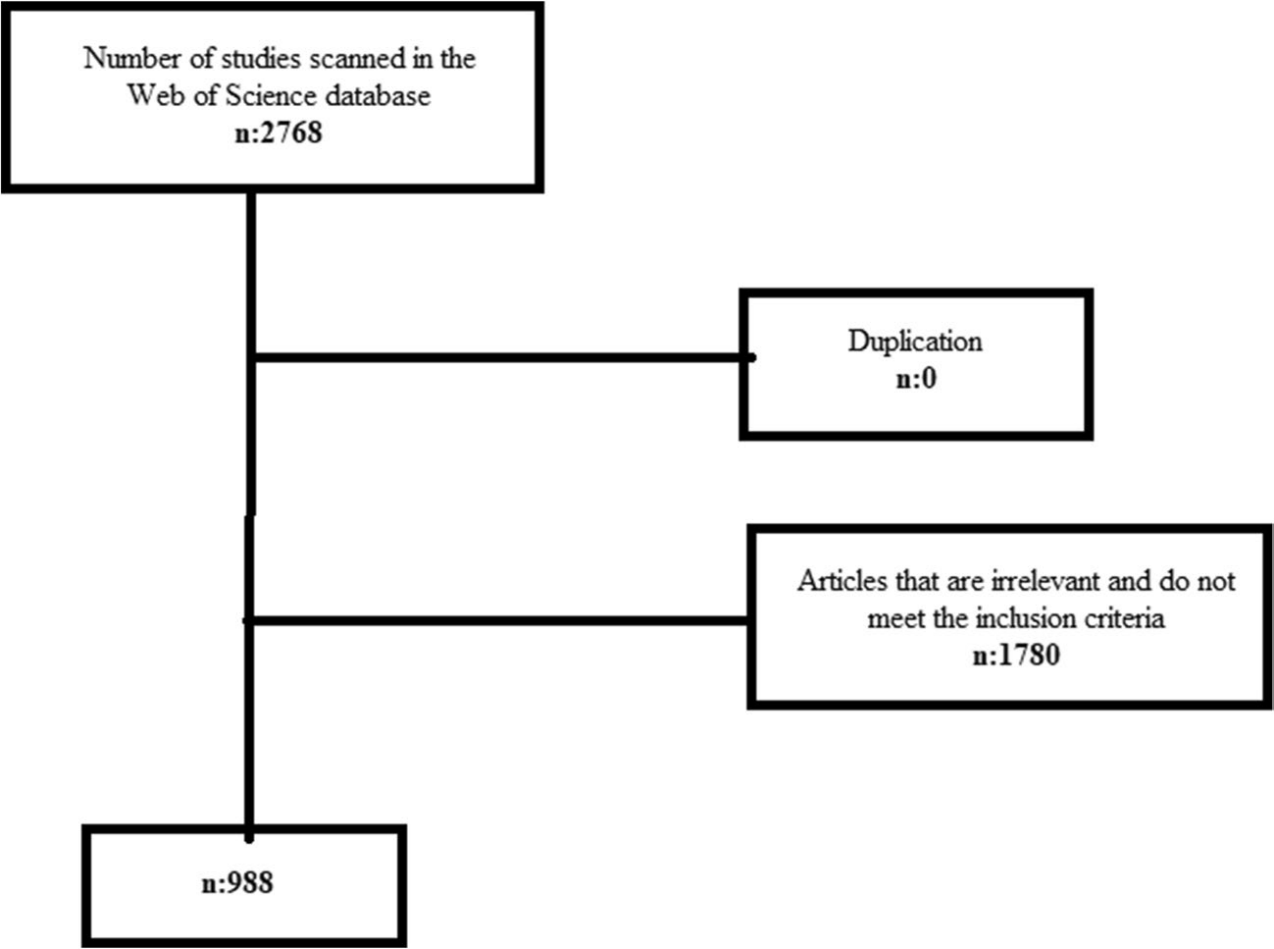
Publication selection flow diagram

Flow diagram of the scans is given in Fig. 1. A total of 988 articles could not be included in the research due to language- and publication-related reasons.

### Publication Numbers and Citation Analysis by Year

**Fig. 2:**
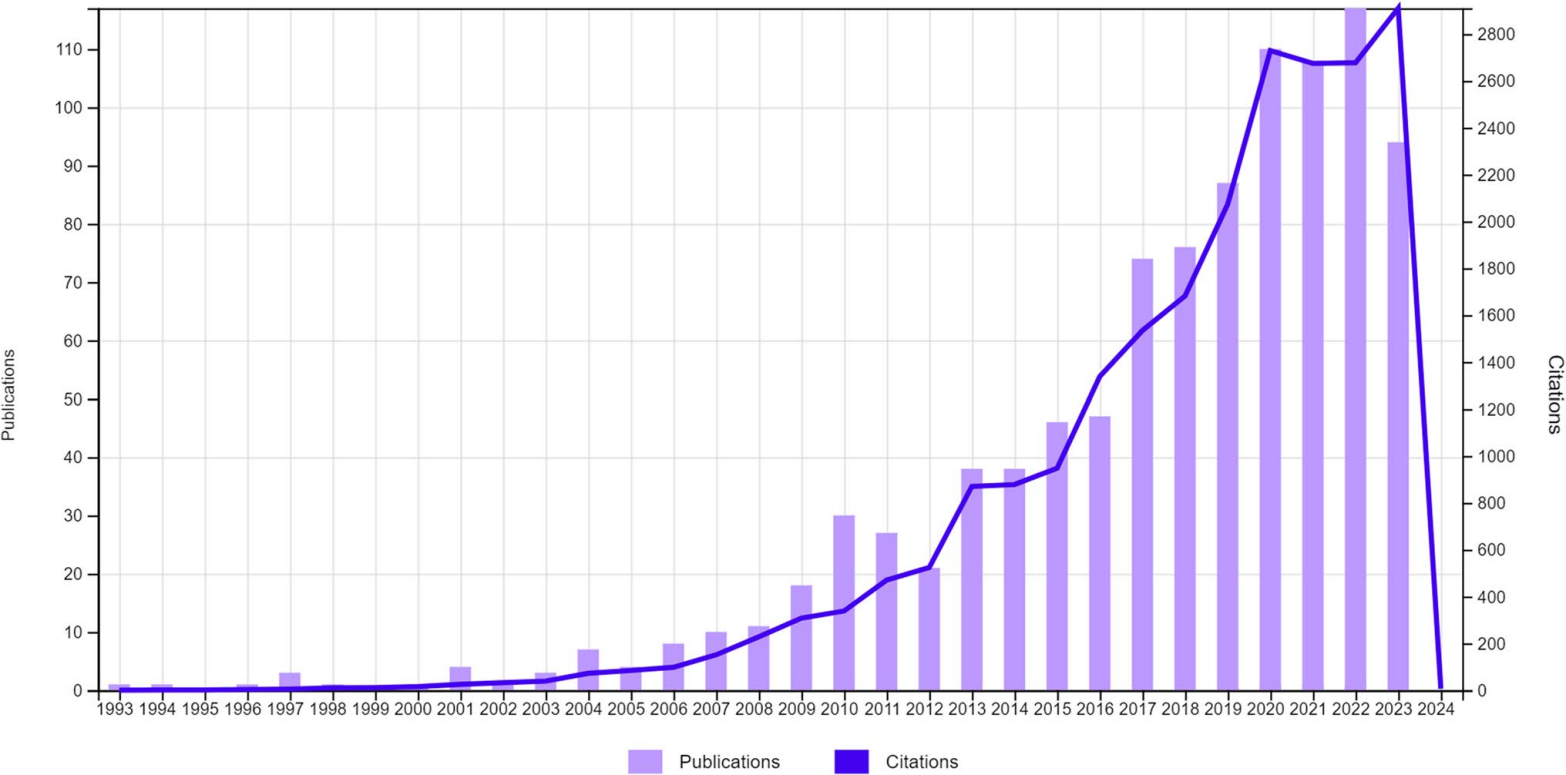
Publications and citations over time

The trends of publications and citations related to weight gain in bariatric surgery are given in Fig. 2. Accordingly, 988 articles published between 1993 and 2023 were reached. The number of publications from the year of the first publication to 2023 showed a surging trend. The highest number of publications (118 articles) was in 2022, while the year with the highest number of citations was 2020 (2729 citations).

A total of 10,533 sources were cited in 988 articles conducted between 1993 and 2023. The articles included in the research were referred to a total of 22.443 times. The average number of citations was 23.12. The average number of citations per year was 745.27. When the number of citations by year was examined, the most citations (*n* = 2670) were made in 2020, and 10.75% of the studies had not received any citations yet. The article titled “Weight gain after short- and long-limb gastric bypass in patients followed for longer than 10 years” was the most cited publication with 488 citations. The top ten articles by the number of citations are given in Table 2.

**Table 2:** The top 10 articles according to the number of citations

Title	Authors	Source title	Publication year	Total citations	Average per year
Weight gain after short- and long-limb gastric bypass in patients followed for longer than 10 years	Christou, Nicolas V; Look, Dider; MacLean, Lloyd D	Annals of Surgery	2006	488	27.11
Long-term weight regain after gastric bypass: A 5-year prospective study	Magro, Daniela Oliveira; Geloneze, Bruno; Delfini, Regis; et al	Obesity Surgery	2008	452	28.25
Effects of Sleeve Gastrectomy in Neonatally Streptozotocin-Induced Diabetic Rats	Wang, Yan; Yan, Lingling; Jin, Zhendong; et al	Plos One	2011	402	31.15
Seven-Year Weight Trajectories and Health Outcomes in the Longitudinal Assessment of Bariatric Surgery (LABS) Study	Courcoulas, Anita P; King, Wendy C; Belle, SH; et al.	JAMA Surgery	2018	380	63.17
Sleeve Gastrectomy as Sole and Definitive Bariatric Procedure: 5-Year Results for Weight Loss and Ghrelin	Bohdjalian, A; Langer, FB; Shakeri-Leidenmühler, S; et al	Obesity Surgery	2010	363	25.93
Laparoscopic sleeve gastrectomy—Influence of sleeve size and resected gastric volume	Weiner, RA; Weiner, S; Pomhoff, I; et al	Obesity Surgery	2007	304	17.88
Re-emergence of diabetes after gastric bypass in patients with mid- to long-term follow-up	DiGiorgi, M; Rosen, DJ; Choi, JJ; et al	Surgery for Obesity and Related Diseases	2010	214	15.29
Reflux, Sleeve Dilation, and Barrett’s Esophagus after Laparoscopic Sleeve Gastrectomy: Long-Term Follow-Up	Felsenreich, DM; Kefurt, R; Schermann, M; et al	Obesity Surgery	2017	190	27.1
Laparoscopic sleeve gastrectomy as a single-stage procedure for the treatment of morbid obesity and the resulting quality of life, resolution of comorbidities, food tolerance, and 6-year weight loss	D’Hondt, M; Vanneste, S; Pottel, H; et al	Surgical Endoscopy and Other Interventional Techniques	2011	186	14.31
One Thousand Consecutive Mini-Gastric Bypass: Short- and Long-term Outcome	Noun, R; Skaff, J; Riachi, E; et al	Obesity Surgery	2012	185	15.42

### Publication Analysis of Journals

A total of 190 journals made publications related to weight gain in bariatric surgery. According to the analysis results, the top three published journals were *Obesity Surgery*, *Surgery for Obesity and Related Diseases*, and *Surgical Endoscopy and other Interventional Techniques*. *Obesity Surgery* journal had the 38.7% of the articles and 383 publications in total.

### Publication Analysis of Countries and Institutions

The country with the most publications on the subject was the USA with 313 articles. Brazil and France were the next, while Turkey was the 21st with 17 publications. The institution with most relevant publication was “Harvard University” with 50 publications. It was followed by “Brigham Women’s Hospital” and “Egyptian Knowledge Bank.”

### Keyword and Abstract Analysis

**Fig. 3:**
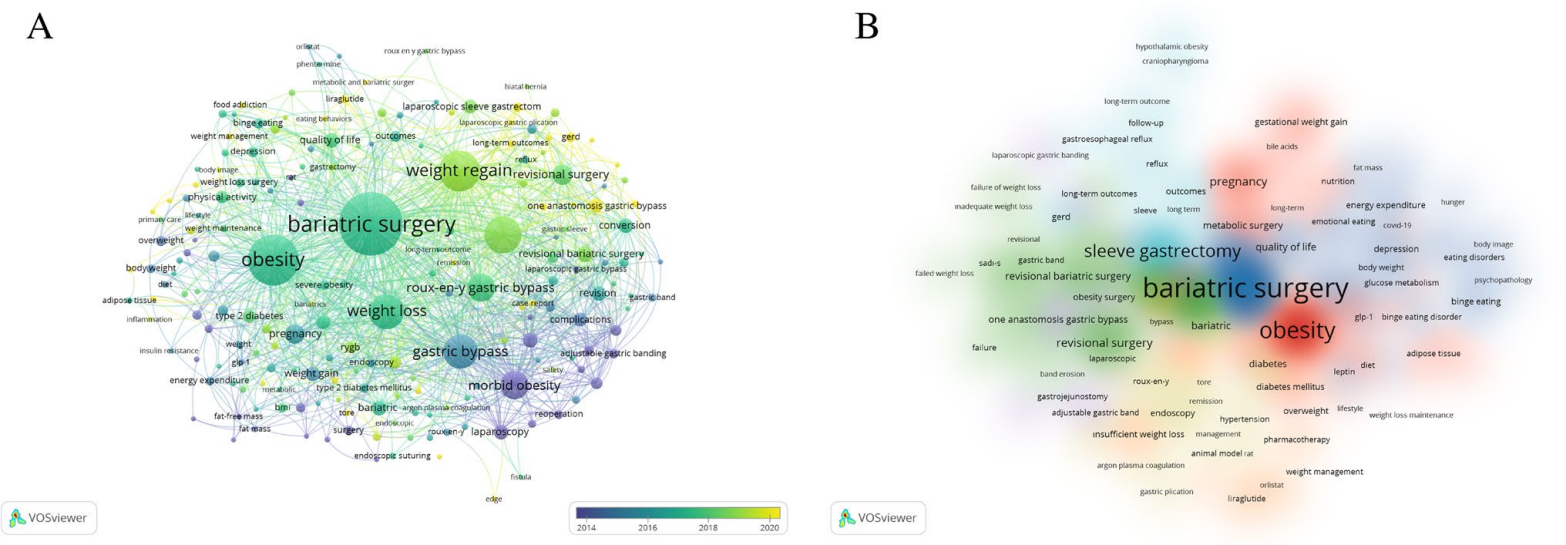
Analysis of the co-occurrence of keywords. **A** Visualization, **B** Density

Co-word analysis was performed to reveal the conceptual structure of the studies. It was used to determine the most important and latest topics in the field and their relationships [27–30]. The co-occurrence analysis of the keywords used in 988 articles was given (Fig. 3). The first three keywords showing co-occurrence were “bariatric surgery” (*n*, 652), “obesity” (*n*, 431), and “weight regain” (*n*, 283).

**Fig. 4:**
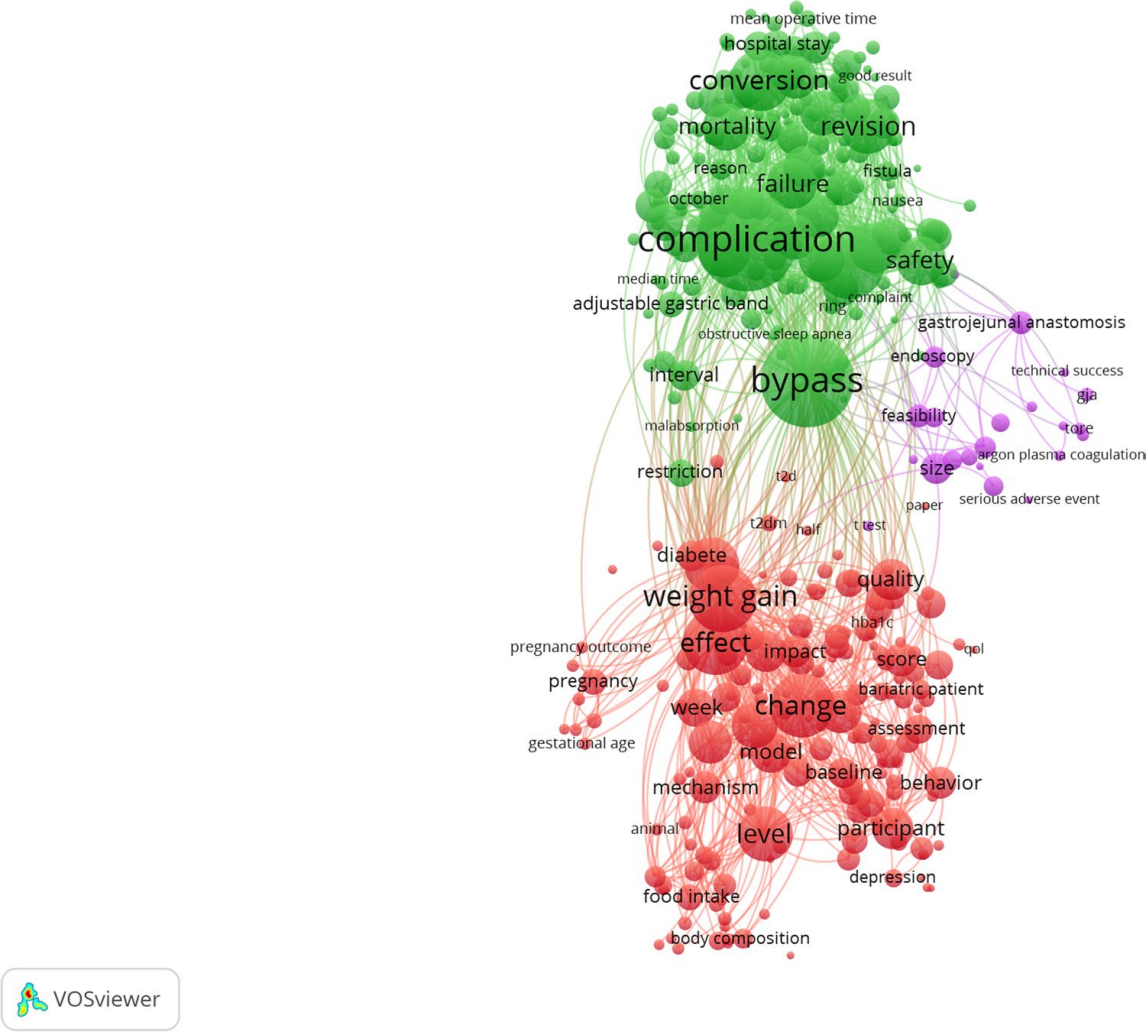
The clustering of the words included in the article abstracts

The mostly used common words in the abstracts of the articles were analyzed. As a result of the analysis of 10 words in abstracts, 350 words were found to be the mostly used. As shown in Fig. 4, the words were divided into three clusters. The clusters mostly consisted of concepts related to types of surgery, weight gain, patient profile, and surgery results.

### Author Performance Analysis

When the most published authors on the subject were examined on VOSviewer, “Christopher C. Thompson” with 30, “Pichamol Jirapinyo” and “Gerhard Prager” with 18 ranked in the top three. The most cited authors were “Herman Toplak” (1040), “Volkan Yumuk” (1040), and “Gerhard Prager” (965), respectively.

Co-author analysis showed the contribution of two or more authors. The authors reflected the cooperation between institutions and countries. The co-author network of this article consisted of 317 authors who had published at least 3 articles each. The authors created a total of 22 common clusters. The largest clusters consisted of 33, 26, and 24 authors, respectively. The highest total connection strengths of the clusters were 634, 451, and 436. The high total connection strength is an indicator of a strong cooperation. The connection strength of the cluster with Thompson and Jirapinyo was 634, while it was 472 with Prager.

**Fig. 5:**
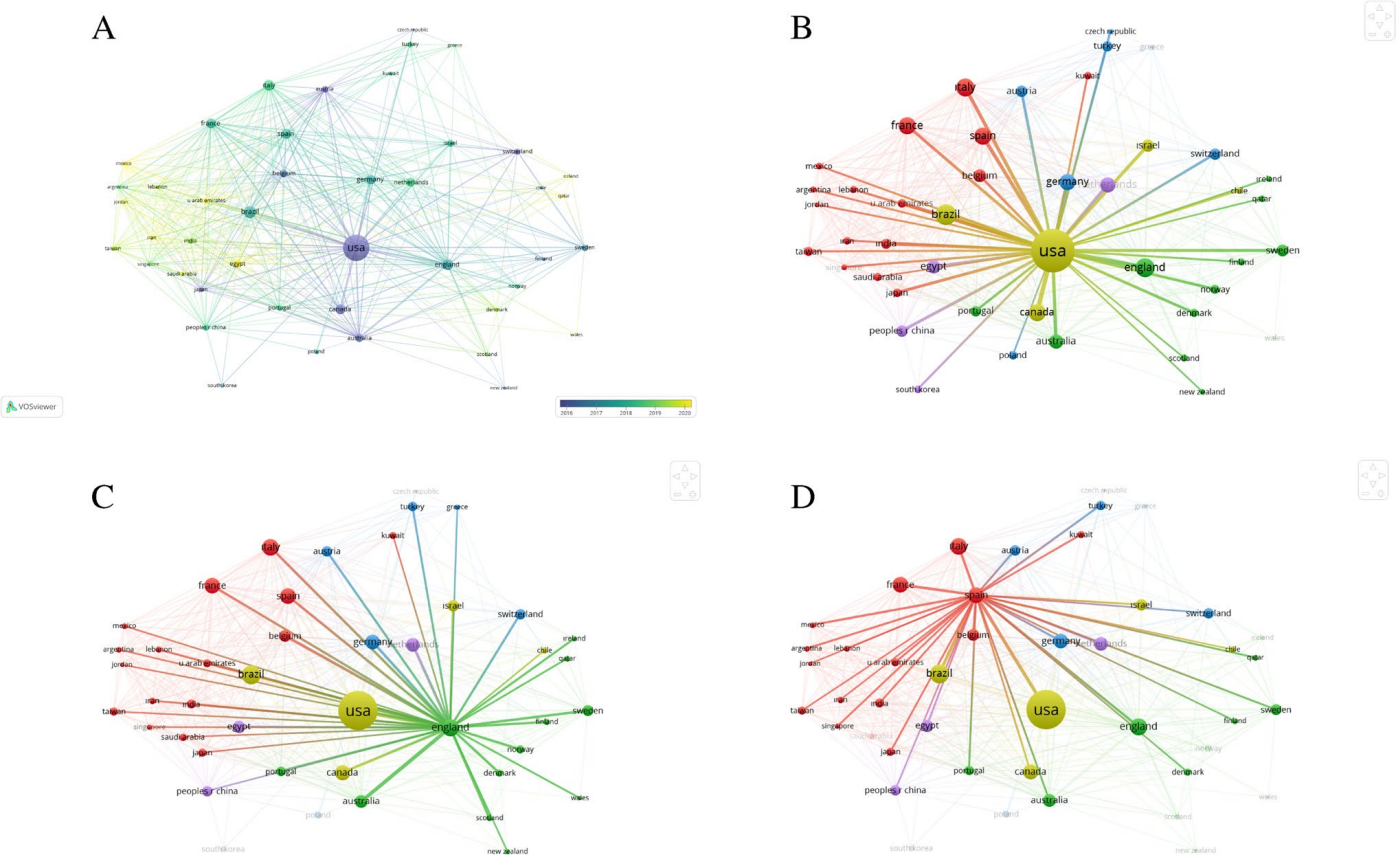
**A** Network visualization of country associations, **B** countries in cooperation with the USA, **C** countries in cooperation with Brazil, and **D** countries in cooperation with the UK

The cooperation network analysis of the countries was performed to determine the cooperation of the researchers with different countries. Each round in the network represents a country, while the link represents inter-country business associations. Network visualization is given in Fig. 5. The USA was the country with the most cooperation, followed by the UK and Spain.

### Co-citation Analysis of Journals

**Fig. 6:**
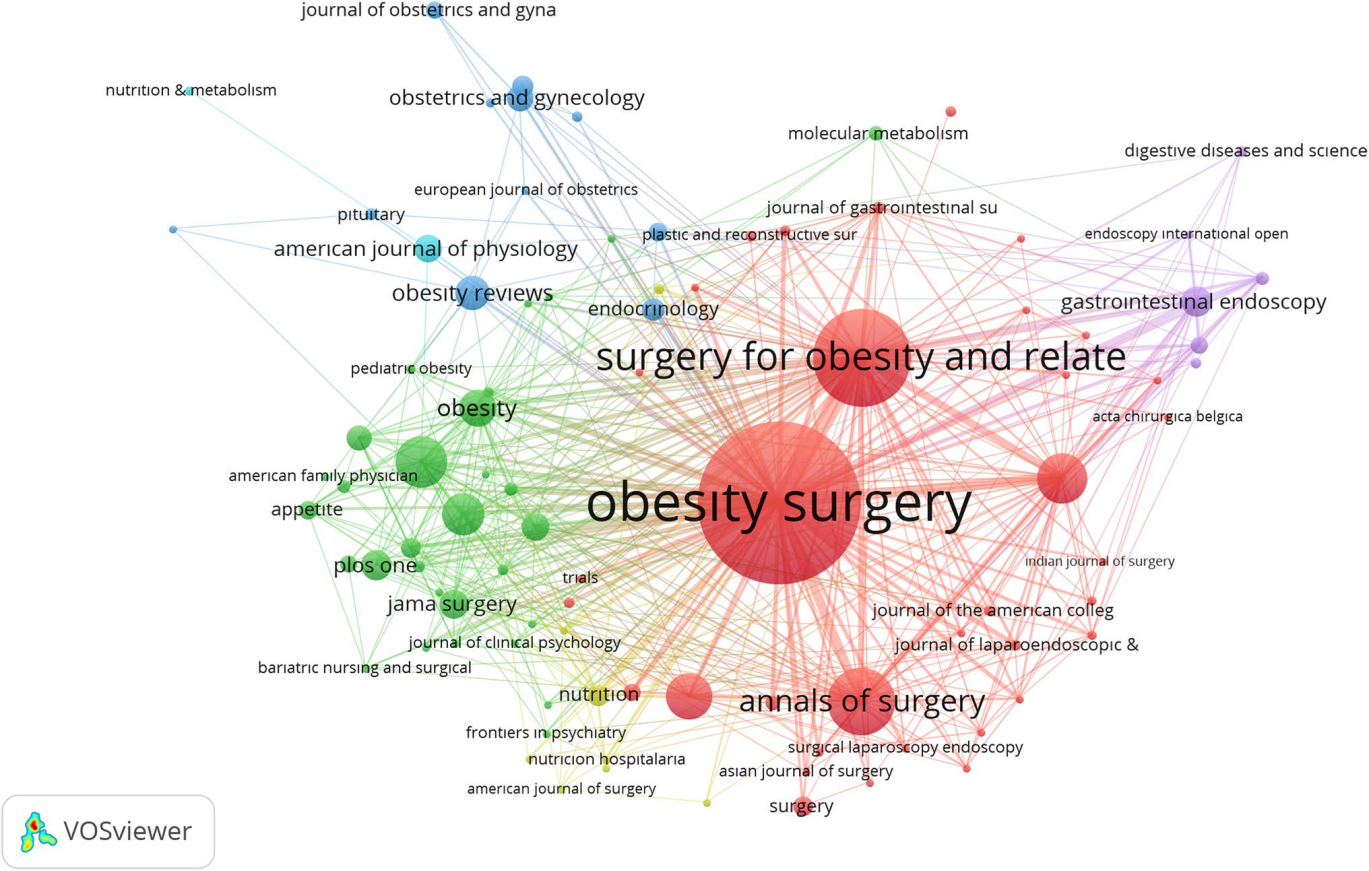
Co-citation analysis of journals

The co-citation analysis is used to determine the network of relationships between the journals that receive the most citations. It shows the frequency of two publications being cited together. In the study, a co-citation analysis of journals was performed based on the reference of 988 articles. The co-citation network had 6 clusters, and each journal had more than 10 citations consisting of 94 journals. The journal with the largest co-citation network was *Obesity Surgery* with 12,361 citations. It was followed by *Surgery for Obesity and Related Diseases* (4804 citations) and *Annals of Surgery* (1108 citations) (Fig. 6).

## Discussion

Bibliometric analysis is a macro-oriented method used to map the intellectual structure of a field, the developments, and the relationships between authors-subjects-studies. It offers a broad perspective on the structure, dynamics, evolution of the field, and the relationship between authors, studies, and words/concepts [31].

Weight regain after bariatric surgery is one of the most important factors that adversely affects the long-term success of surgery. After bariatric surgery, 20–30% of individuals fail to achieve the targeted weight loss or may start to regain weight 18–24 months after surgery [32, 33]. Studies indicate an increasing number of people who regain weight after surgery [2, 34, 35]. It is difficult to report due to the lack of a standard criterion and the need for long-term follow-up [10]. This research is an overview of weight gain after bariatric surgery.

All developments related to bariatric surgery from the past to the present indicate that patients experience weight gain after surgery, which has become an important issue for the patient group. As shown in Fig. 2, the number of publications, which was only one in 1993, has reached 118 in 2022 with an increasing momentum. It proves that weight gain after surgery is an important problem. The increase in the number of obese individuals and surgeries are also other reasons for increasing number of publications.

In this study, important articles, influential authors and journals, citations, and concepts were identified. No studies being found to determine the research trends in bariatric surgery and weight gain and analyze the contents constitutes the original aspect of our study.

According to the results of bibliometric analysis, the number of studies conducted on weight gain in bariatric surgery showed an increase over time. The highest number of articles on weight regain was 110 and 118 in 2020 and 2022, respectively. It is considered that the need for further research on weight gain after bariatric surgery and increased productivity related to the field may be effective in increasing number of publications.

The articles on weight gain were published by authors from 67 different countries. The countries with the most publication was the USA, Brazil, the UK, Italy, and France, respectively. The USA was by far the most productive country among 61 countries. According to IFSO 2022 data, the number of bariatric surgeries in the USA was 170,055 [36]. These results are expected since it performs the greatest number of surgeries and individuals with obesity. Other bibliometric studies also support this result [37–39].

It was found in this study that a total of 2167 institutions contributed to the field, and the most productive institutions were “Harvard University,” “Brigham Women’s Hospital,” and “Egyptian Knowledge Bank.” Non-university institutions also had studies related to the field [40]. The Egyptian Knowledge Bank has been the largest library archive in Egypt since 2016. It includes numerous national and international publishing houses within its scope [41].

The 988 studies included in the analysis were published in 190 different journals. The highest number of publications were made in the journal *Obesity Surgery*, the official publication of the International Federation for the Surgery of Obesity and metabolic disorders (IFSO), and the British Obesity & Metabolic Surgery Society (BOMSS). The impact factor of the journal published monthly in 2022 was 2.9, and the number of downloads was 1,115,070 [10, 32–36, 40, 42, 43]. This journal being the leading journal in the field proves its performance and quality [44]. This result shows similarities with other studies [37, 38]. In a study investigating the publication trends related to bariatric surgery, *Obesity Surgery* was also mentioned as the most published and most influential journal in the field [39]. It may be recommended that researchers prefer leading journals and follow the developments through them.

Citation analyses are based on the use of the studies by the authors by other authors and provide information about the effectiveness of the study [31]. Studies that receive more citations have a greater impact on the field. It can also draw a roadmap for authors for reading. The co-citation analysis is the frequency of two studies being cited together [31]. The co-citation is most often included in the journals *Obesity Surgery* (12,361 co-citations), *Surgery for Obesity and Related Diseases* (4804), and *Annals of Surgery* (2368) (Fig. 6). It is seen that journals with a strong co-citation network are also strong in the number of citations.

The co-occurrence analysis examines the relationship between the concepts and the words. The keywords in the studies included in the analysis included “bariatric surgery” (*n*, 652), “obesity” (*n*, 431), and “weight regain” (*n*, 283). The words formed three clusters as the types of surgery, weight gain, and metabolic consequences. These clusters show the dominant research topics and the relationships between them. The clusters revealed as a result of the analysis overlapped with the subject clusters in other studies [38, 45].

There are some limitations in the study. Firstly, the WoS database was used, and other databases were not included. In addition, only articles were included, and books, book chapters, and grey literature publications were excluded. In the sample selection, only English articles were included. Publications in other languages were excluded. A more comprehensive analysis, including different languages and other publications, can be performed in the future studies.

## Conclusion

Weight regain after bariatric surgery is becoming an increasingly prevalent issue in contemporary times. Weight regain is an important issue that needs to be addressed because it negatively affects the lives of individuals, reduces the likelihood of success of surgical treatment, and puts an additional burden on the healthcare system. Through a bibliometric analysis, a comprehensive assessment of the field of weight regain in bariatric surgery has been conducted. Emerging trends have been identified, and contributors such as authors, journals, and institutions making the most impact in this field have been highlighted. The contents and tendencies of articles have been scrutinized. As a result, a perspective on the significant issue of weight regain has been presented from the standpoint of countries, institutions, and authors. In light of this, efforts should be directed towards preventive measures at the country and institution levels regarding weight regain. Additionally, the escalating number of publications underscores the growing need for addressing this issue.

## Data Availability

The following licenses and limitations apply to the data used in this study: The Web of Science, which is available to academic researchers in Turkey under license from the Joint Information Services Committee and in other nations through separate licensing arrangements, was the source of original data when specified. Please contact https://clarivate.com/webofsciencegroup/solutions/web-of-science/contact-us/ with any requests for access to these datasets.
